# Presentation and Management of High-Grade Pancreatic Injuries: Case Series

**DOI:** 10.3390/reports9030311

**Published:** 2026-09-14

**Authors:** Khalid Ahmed, Ahmad Kloub, Ayman El-Menyar, Mohammad Asim, Hassan Al-Thani, Ruben Peralta, Sandro Rizoli

**Affiliations:** 1 Department of Surgery, Trauma Surgery, Hamad Medical Corporation (HMC), Doha P.O. Box 3050, Qatar; kahmed11@hamad.qa (K.A.); akloub@hamad.qa (A.K.); althanih@hotmail.com (H.A.-T.); rperaltamd@gmail.com (R.P.); srizoli@hamad.qa (S.R.); 2 Department of Surgery, Trauma Surgery, Clinical Research, Hamad Medical Corporation (HMC), Doha P.O. Box 3050, Qatar; masim1@hamad.qa; 3 Department of Internal Medicine, Weill Cornell Medical College, Doha P.O. Box 24144, Qatar; 4 Department of Surgery, Universidad Nacional Pedro Henriquez Urena, Santo Domingo 10100, Dominican Republic

**Keywords:** pancreas, high-grade injury, trauma, abdominal injuries, drainage, pancreatectomy

## Abstract

**Background**: Trauma surgeons face a challenge in the management of high-grade pancreatic injuries (HGPIs), as optimal strategies remain controversial. HGPIs are uncommon and difficult to manage due to dynamic trauma physiology and complications from the injury and management. We aimed to evaluate HGPI presentations, management, and outcomes. **Methods**: This retrospective study included 13 patients with HGPIs (American Association for the Surgery of Trauma [AAST] grade III–V) who underwent interventions between 2017 and 2024. **Results**: There were 13 cases with HGPIs (five Grade III, four Grade IV, and four Grade V). Liver injury was the most common associated organ injury (61.5%), followed by splenic injury (46.2%). Distal pancreatectomy was the most frequently performed operation, with either concomitant splenectomy (53.8%) or spleen preservation (23.1%). Severe postoperative infection was observed in this cohort, with sepsis occurring in 69.2% of patients and peripancreatic collections noted in 76.9% and intra-abdominal infection in 61.5%. Other postoperative complications included ileus (61.5%), pancreatic fistula (30.8%), and diabetes (15.4%). **Conclusions**: This case series demonstrates that high-grade pancreatic injuries are associated with substantial morbidity, along with prolonged hospitalization. A higher Abbreviated Injury Scale (AIS) among HGPIs is associated with greater overall injury severity and higher rates of major complications. Clinicians should consider prioritizing early drainage procedures, particularly in Grade IV and V injuries. Multicenter and larger-sample-size studies are required.

## 1. Introduction

Pancreatic trauma is an uncommon entity occurring in approximately 0.2–0.3% of all trauma patients [1]. The ductal injury is the principal cause of pancreatic-specific morbidity and mortality leading to significant disease burden from complications including include acute hemorrhage, pancreatic leaks, abscesses, fistulae, and pancreatitis [2]. Historically, injuries to the pancreas were described by their location, and the American Association for the Surgery of Trauma stated that higher-grade injuries correlate with higher mortality and complications [3].

Pancreatic injuries are rarely isolated and are typically accompanied by other intra-abdominal injuries, further complicating detection with conventional imaging techniques. Imaging findings can be subtle, especially when studies are obtained within the first 12 h after injury. Specific signs of pancreatic trauma include pancreatic fractures or lacerations, active hemorrhage, and parenchymal contusion, edema, or hematoma. At the same time, nonspecific findings may consist of peripancreatic hemorrhage or surrounding fat stranding [4]. The core principles shared by both the Eastern Association for the Surgery of Trauma (EAST) and the Western Trauma Association (WTA) emphasize individualized management of pancreatic injuries. Given the variation in management strategies recommended by these organizations, it is important to directly compare their practical applications and effectiveness in real-world scenarios [5,6]. By juxtaposing these recommendations with local outcomes, we aim to evaluate the presentations, management, and outcomes of high-grade pancreatic injuries.

## 2. Materials and Methods

We conducted a retrospective case series for adult patients admitted between 2017 and 2024 with high-grade (AAST grade III–V) pancreatic injuries who underwent surgical management. Data were retrieved from a well-validated prospective national trauma registry (QNTR) from Hamad Trauma Center in Qatar. The QNTR contributes quarterly data to the American College of Surgeons (ACS) Committee on Trauma’s National Trauma Databank (NTDB) and participates in the Trauma Quality Improvement Program (TQIP) [7]. The collected data encompassed demographics, injury mechanism, physiologic parameters, associated injuries, injury severity scores such as injury severity score (ISS) and aAbbreviated injury scale (AIS), pancreatic injury grade, imaging, operative details, complications, and outcomes. Pediatric patients, those with low-grade (I–II) pancreatic injuries, and patients managed nonoperatively were excluded.

Only adult patients (age ≥ 18 years) were eligible for inclusion; patients younger than 18 years at the time of injury were classified as pediatric and excluded regardless of injury grade or management. All included patients sustained trauma requiring surgical intervention; both blunt (motor vehicle collision, pedestrian-struck, motorcycle collision, fall, assault, and blast/explosion) and penetrating (gunshot and stab wound) mechanisms were eligible, and no trauma mechanism was excluded a priori. Patients with isolated non-traumatic pancreatic pathology (e.g., pancreatitis unrelated to injury) were not included. All operative and nonoperative therapeutic decisions were made by the on-call attending trauma/general surgeons, all of whom held certified board qualification in trauma and general surgery with a minimum of 5 years of post-fellowship attending experience in a level 1 trauma center; a total of 7 attending surgeons were involved in the operative management of the 13 cases included in this series, in keeping with our institution’s multidisciplinary trauma call rotation.

The AIS defines injury severity by body region; it ranges from 1 to 6, representing minor, moderate, serious, severe, critical, and non-survivable injuries, respectively. The AIS scores of the three most severely injured body regions are squared and added to estimate the ISS, which provides an overall score for polytrauma. The ISS ranges from 0 to 75; 1–8 is major, 9–15 is moderate, 16–24 is serious, 50–74 is critical, and 75 is non-survivable [8].

Management followed a standardized institutional damage-control approach rather than a fixed step-by-step protocol, in line with EAST/WTA guidance that pancreatic trauma management should be individualized [5,6]. In brief, all patients underwent initial resuscitation per Advanced Trauma Life Support principles; hemodynamically unstable patients or those with peritonitis proceeded directly to exploratory laparotomy, while stable patients underwent contrast-enhanced CT (supplemented by MRI/MRCP or ERCP when duct integrity was in question) to define injury grade and duct status before intervention.

**Figure 1:**
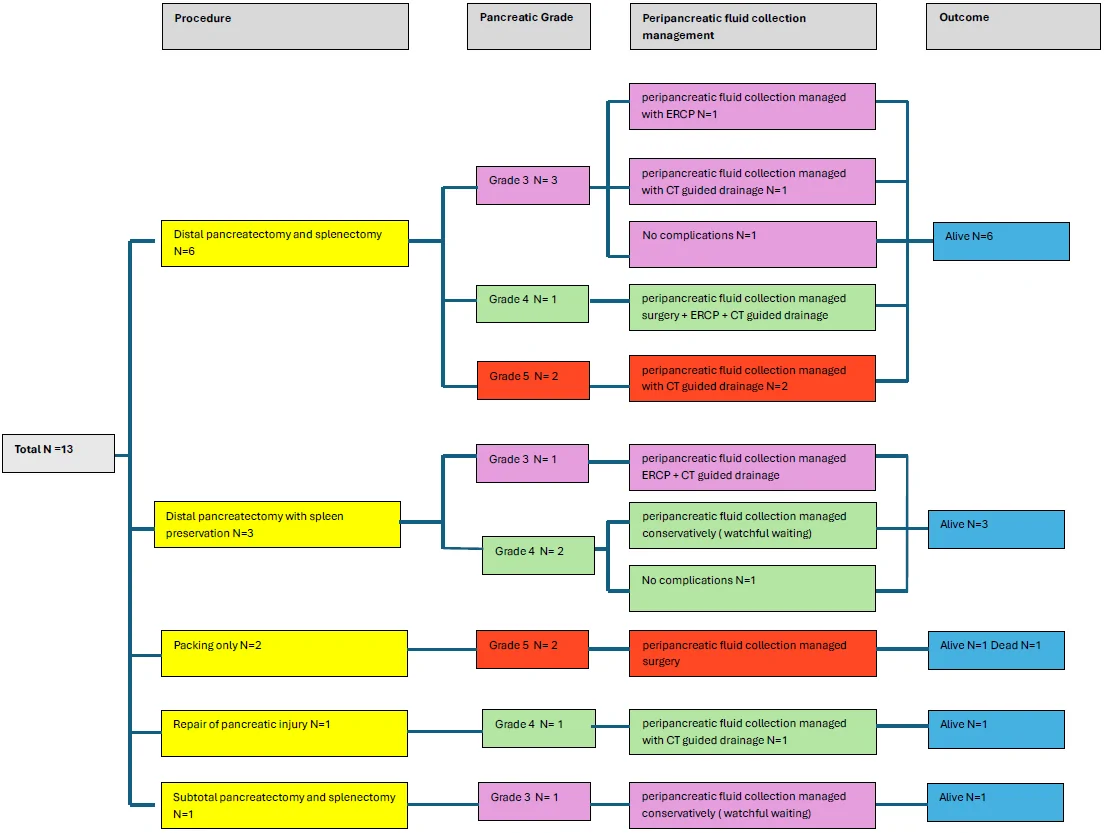
Study design, management and outcomes based on the pancreatic injury grade.

At laparotomy, the decision between resection (distal pancreatectomy, with or without splenectomy) and drainage/repair/packing was guided by three factors applied consistently across cases: (i) anatomical location of the injury relative to the superior mesenteric vessels (injuries to the left of the vessels were considered amenable to distal pancreatectomy, whereas injuries at or to the right of the vessels, i.e., the head/uncinate process, were managed with drainage, packing, or repair rather than resection); (ii) main pancreatic duct integrity, assessed intraoperatively or via ERCP/MRCP; and (iii) physiological reserve, with damage-control packing and staged relaparotomy reserved for hemodynamically unstable or coagulopathic patients irrespective of injury location. Wide peripancreatic drainage was placed in all resection and repair cases; postoperative Octreotide, staged CT-guided drainage of collections, and targeted antibiotic therapy guided by culture data were used adjunctively as detailed below and summarized in Figure 1. This algorithm is consistent with our unit’s broader trauma protocol and was applied uniformly to all 13 cases, which explains the apparent heterogeneity of procedures for a given injury grade.

Data were presented as number, percentage, mean (standard deviation; SD), median and range whenever applicable; otherwise, no inferential statistics were required because of the small number of cases. The study received approval from the Medical Research Center (MRC-01-25-179) at the Hamad Medical Corporation, Doha, Qatar. No written informed consent was required for this retrospective case series, as data were collected without direct patient contact and with no identifiers linked to the participants. We conducted basic descriptive analyses (median, range, percentages, and averages) where applicable. All data analyses were carried out using SPSS version 22 (Armonk, NY, USA).

## 3. Results

Thirteen patients undergoing surgical management for high-grade (III–V) pancreatic injuries were included. Most were male (84.6%) with a mean age of 29.5 (SD 8.5) years. Blunt trauma predominated (92.3%), and the median Glasgow Coma Scale (GCS) at the Trauma Resuscitation Unit (TRU) was 15 (range 12–15), with normal vital sign limits.

Table 1 shows demographics, clinical features, associated injuries, and complications in surgically managed patients.

Patients had severe multi-system trauma (mean ISS 21.2 (SD 8.3)) and pancreatic injury (mean AIS 3.9 (SD 0.9); abdominal AIS 3.8 (SD 1.1)). Table 2 summarizes the clinical characteristics of the 13 cases. The pancreatic head was most involved (38.5%), followed by lacerations (30.8%), with complete transections and body injuries each at 23.1%. Three patients had duodenal injuries, of which one had complete duodenal transaction while the other two had retroperitoneal duodenal hematomas only. Liver (61.5%) and spleen (46.2%) were the most frequent associated injuries. Rib fractures and chest injuries occurred in 38.5%, while bowel, colon, mesentery, and spine injuries each occurred in 23.1%.

**Figure 2:**
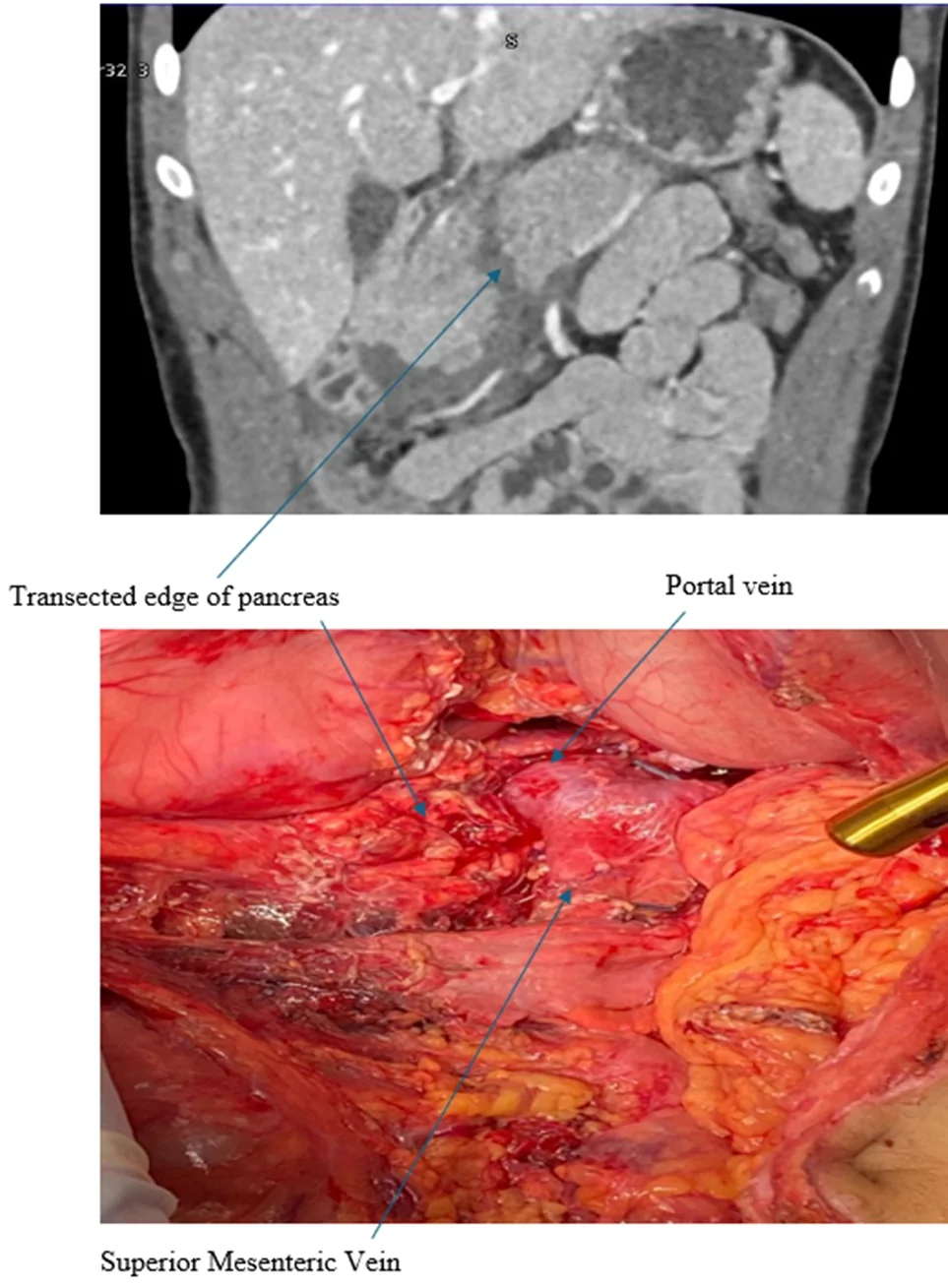
Grade III pancreatic injury (**Upper**: CT scan and **Lower**: intraoperative).

**Figure 3:**
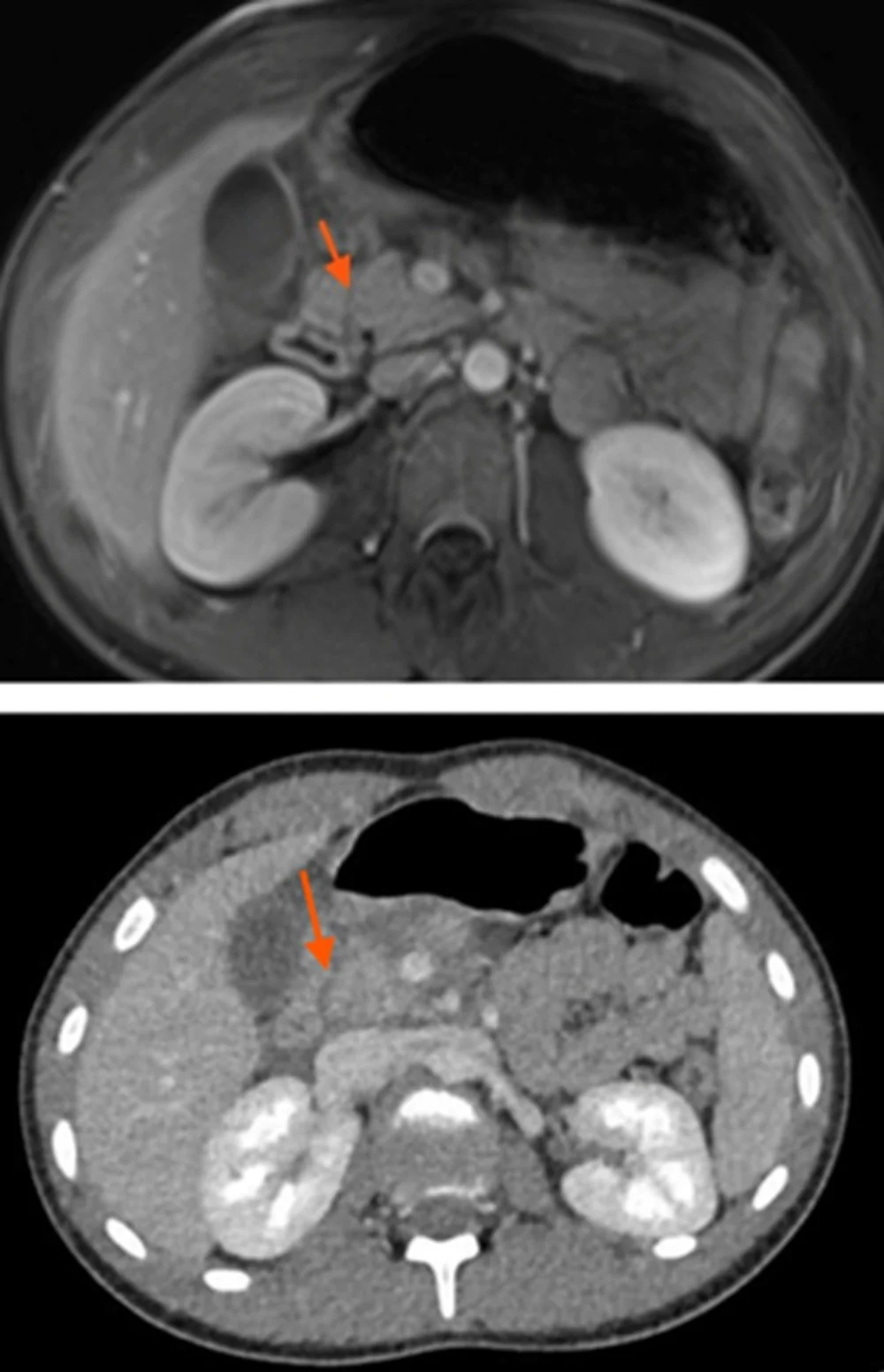
(**Upper**): MRI description of the complete transection of the neck of the pancreas, right of the mesenteric vessels. (**Lower**): Axial view of abdominal CT scan depicting Grade IV complete transection of the pancreas at the neck of the gland (Arrows indicate a pancreatic injury).

Although the pancreatic head/neck region was the most frequently injured site (38.5%), this did not translate into head-directed resections (e.g., pancreaticoduodenectomy) because, as detailed in the Methods section, the operative strategy was dictated by injury location relative to the superior mesenteric vessels and by duct status rather than by injury site alone. In the five patients with head or neck involvement, the disrupted parenchyma/duct was consistently located at or just to the left of the mesenteric vessels, allowing the injury to be managed by distal pancreatectomy with resection extending up to, but not beyond, the neck, thereby preserving the head and avoiding the substantially higher morbidity and mortality of pancreaticoduodenectomy. None of our patients had a duct injury isolated to the head/uncinate process itself that mandated a Whipple procedure; those with retroperitoneal duodenal hematoma or head contusion without ductal disruption were managed with drainage, packing, or repair alone. This explains why distal pancreatectomy remained the dominant operation despite the head being the most commonly injured region. Figure 2 and Figure 3 are examples of high-grade pancreatic injuries.

A striking pattern in this cohort was the high burden of infectious and fluid-related complications following intervention. The most common surgical procedure was distal pancreatectomy with splenectomy (53.8%), followed by distal pancreatectomy with spleen preservation (23.1%) and packing/repair (23.1%). Peripancreatic collections were the most frequent pancreatic-specific complication (76.9%), particularly linked to distal pancreatectomy procedures, where fluid accumulation was common. High rates of systemic infection were observed: sepsis (69.2%) often followed resection surgeries, while intra-abdominal infection (61.5%) was frequently noted post-repair operations. Other common complications included postoperative ileus (61.5%), predominantly after pancreatectomy with splenectomy, and blood transfusion (46.2%), which was necessary in cases with significant blood loss from extensive operations. Pancreatic fistula (30.8%) was most commonly observed after resection, suggesting an association between surgical procedure type and complication rates. Figure 1 summarizes the management of high-grade pancreatic injuries. Table 3 shows complications and outcomes in surgically managed patients.

Median hospital stay was 25 days (range 1–107), ICU stay 7 days (1–36), and ventilatory support 8 days (1–23). Overall mortality was 7.7% (one patient). Most patients (76.9%) were discharged stable, while 23.1% were discharged with a drain, reflecting ongoing recovery from complex collections or fistulae. Table 4 describes the three higher injury grades.

Grade III Injuries (five patients)

Surgical management included three distal pancreatectomies with splenectomy, one distal pancreatectomy with spleen preservation, and one subtotal pancreatectomy with splenectomy. Of the patients, 40% required blood transfusions, 60% developed sepsis, and 60% had peripancreatic collections. One patient developed a pancreatic fistula. Management included CT-guided drainage, ERCP, and conservative measures. Four patients were discharged in stable condition, and one had a drain in place. No pancreatitis, pseudocysts, abscesses, or pancreatic insufficiency occurred.

Grade IV Injuries (four patients)

Three patients underwent distal pancreatectomy, while one underwent surgical repair. Postoperative complications were common, with peripancreatic fluid collections in 75% and sepsis in 50% of patients. Management ranged from conservative care to CT-guided drainage and ERCP. No cases of pancreatic necrosis or post-traumatic diabetes occurred, and most patients were discharged in stable condition.

Grade V Injuries (four patients)

Surgical management included distal pancreatectomy with splenectomy and damage-control “packing only” approaches. Mortality strongly correlated with surgical intensity; the female patient, who underwent 10 repeat laparotomies, was the sole fatality, while survivors required 0–3 repeat procedures. Postoperative complications were universal: 100% developed sepsis, 75% had peripancreatic collections and enterocutaneous fistulas, with additional morbidities including pancreatic necrosis, acute kidney injury, and one case of post-resection diabetes. Survivors faced prolonged recoveries involving CT-guided drainage, Octreotide therapy, and multiple readmissions, highlighting the high morbidity of severe pancreatic trauma.

## 4. Discussion

High-grade pancreatic injuries are rare and challenging to manage. Unlike our previous study covering all injury grades, this series focuses on surgically managed grade III–V injuries in a predominantly young, male trauma population [9]. This cohort’s demographic profile aligns with trends observed in national trauma registries, where young males are more frequently represented in cases of high-grade injuries. This consistency may lend weight to external validity and generalizability; however, the number of patients remains a limit for study generalizability. Similar predominance of young male patients and blunt mechanisms has been reported in other recent single- and multi-institutional pancreatic trauma series [10,11,12], and a large tertiary-center analysis has likewise emphasized that penetrating and blunt pancreatic injuries differ in associated-injury pattern and morbidity, further supporting the external validity of our cohort despite its size [11].

Guidelines recommend nonoperative care for low-grade injuries but differ for high-grade cases [5,6]. The WTA emphasizes duct integrity and location to guide resection or repair, while EAST generally favors surgical intervention for severe injuries. Both recommend individualized, often complex procedures for grade V injuries, such as pancreaticoduodenectomy, tailored to associated injuries and patient stability. Accordingly, surgical management was more prevalent, aligning with EAST’s recommendations for severe injuries, especially in Grades IV and V. The WTA’s emphasis on duct integrity was reflected in the selective approach observed in our Grade III cohort, where we occasionally employed preservation techniques. Notably, our series highlights that management selectively diverged from conventional guidelines and may further refine their practical application. In certain cases of high-grade injury with splenic involvement, we opted for spleen-preserving distal pancreatectomy, even when splenectomy would generally be endorsed by EAST and is commonly performed according to WTA recommendations. This decision was influenced by stable hemodynamics, minimal splenic injury, and the desire to preserve immunological function, and it was associated with favorable patient recovery in our cohort. This example illustrates that individualized surgical strategies, informed by intraoperative findings and patient context, can safely adapt or extend existing guidelines in select scenarios. Overall, this alignment underscores the nuanced decision-making required in high-grade pancreatic trauma, where adherence to guideline recommendations must be balanced with clinical judgment.

In this study, a link was observed between increasing AIS grade and overall trauma severity. The ISS appeared to increase with higher injury grades, and the Abdomen AIS demonstrated escalation from Grade III, IV, toV. These observations suggest that as the AIS grade increases, the complexity and potential life-threatening nature of the injuries also rise. AIS is a region-specific injury description; however, ISS reflects the overall injury burden and emergency care needs for polytrauma [13]. It is worth noting that AIS and AAST organ injury grade, although correlated, are not interchangeable; a recent analysis found meaningful discordance between AIS severity and AAST grade in high-grade solid-organ injury, which should be keptin mind when comparing AIS-based severity data across pancreatic trauma cohorts, including ours [14].

Krige et al. reported age and shock as significant predictors of morbidity [15]. In our study, age, gender, initial GCS, and blood pressure did not differ significantly across higher grades. Pancreatic injuries are commonly associated with other injuries in polytrauma. The small bowel injuries were markedly increased from 0% in Grade III to 50.0% in Grade V, suggesting a higher-energy impact on adjacent retroperitoneal structures. Similarly, chest injuries were highest in Grade V–IV compared to Grade III. Previous studies [9,16] reported splenic injury as the most common organ injury; however, the prevalence of splenic and hepatic injuries was highest in Grade III–IV. These findings are broadly consistent with a recent systematic review of over 14,000 pancreatic trauma patients, which identified shock and sepsis as the strongest independent predictors of mortality and confirmed that AAST grade ≥ 3 injuries carry a substantially higher risk of failed nonoperative management, particularly in patients with hemodynamic instability at presentation [17,18].

The clinical utility of serum amylase and lipase remains controversial, and the WTA [6] recommends that enzyme levels should not be relied upon to either diagnose or exclude pancreatic injury. In our cases, the median serum amylase and lipase levels were elevated but exhibited extremely wide ranges. These findings reinforce the existing skepticism described in the guidelines, as the variability we observed mirrors the caution urged by WTA against depending on these biomarkers for reliable diagnosis.

A multicenter study reported that pancreatic complications increase with injury severity and management intensity, with a higher incidence in Grades IV–V. In contrast, Grade III injuries showed similar complication rates between resection and drainage [19]. In our cohort, pancreatic fistula rates rose from 20% in Grade III to 50% in Grade V, with pancreatic necrosis occurring only in Grade V (25%). Sepsis was present in all Grade V patients, compared to 50–60% in Grades III–IV. Blood transfusions were required in 75% of Grade V patients, compared with 25–40% in lower grades. Median ventilator support and ICU stay were longest in Grade V. The sole mortality occurred in Grade V.

Beyond the choice of resection versus repair, our data highlight that perioperative adjuncts were central to managing these patients and merit explicit discussion. Closed-suction peripancreatic drainage was placed at the index operation in all resection and repair/packing cases, consistent with the very high observed rate of peripancreatic fluid collection (76.9%) and reflecting our low threshold for wide external drainage of the pancreatic bed regardless of duct status, an approach supported by contemporary series showing that routine drainage does not increase, and may reduce, clinically significant collections after pancreatic trauma surgery [11,20]. Peritoneal lavage was performed at the time of damage-control laparotomy in all patients requiring packing or relaparotomy, principally to control contamination from associated hollow-viscus injury rather than as a pancreas-specific intervention, given that source control of concomitant bowel, colonic and biliary injury was the dominant driver of the high intra-abdominal infection rate (61.5%) in this cohort. Empirical broad-spectrum antibiotics were initiated at admission in accordance with our institutional damage-control protocol and were subsequently narrowed according to culture and sensitivity results from intraoperative or drain-fluid samples; antibiotic duration was individualized and extended in patients with confirmed intra-abdominal infection, sepsis, or enterocutaneous fistula. Octreotide was used adjunctively in 46.2% of patients, predominantly those with high-output fistula or persistent collections, to reduce exocrine pancreatic secretion, although its evidence base in traumatic (as opposed to post-pancreatectomy) fistula remains limited. When peripancreatic collections did develop, our default management was staged, a minimally invasive escalation, i.e., CT-guided percutaneous drainage as first-line therapy, with ERCP and endoscopic stenting reserved for collections with a demonstrable ductal communication, and surgical re-intervention reserved for failure of these measures or ongoing sepsis, an approach that mirrors the stepwise strategy advocated in recent multicenter and systematic-review data on high-grade pancreatic trauma [11,17].

To enhance patient outcomes, these recommendations could be considered:

− Early initiation of drainage procedures that can mitigate the risk of peripancreatic collection complications;
− Regular monitoring for signs of infection with a proactive approach to antibiotic treatment that helps in reducing sepsis rates;
− Close observation and intervention for potential fistula formation that can significantly improve recovery trajectories.

### Limitations

The retrospective nature, single-center data, and relatively small sample size are key limitations. Because this was a descriptive case series and formal statistical testing was not performed, we did not report *p*-values. We acknowledge that future analyses with larger sample sizes could further clarify the significance of these associations. Follow-up is required to address the long-term impact of these severe injuries and their early management. However, larger-sample studies that exclusively include high-grade pancreatic injury are scarce, and multicenter prospective clinical studies are needed.

## 5. Conclusions

The high-grade pancreatic injuries (HGPI), are associated with substantial morbidity, prolonged hospitalization, and frequent pancreatic and infectious complications. Increasing the Abbreviated Injury Scale (AIS) correlates with greater injury severity and higher rates of major complications, with the greatest morbidity and mortality observed in Grade V injuries. Clinicians should consider prioritizing early drainage, particularly in Grade IV and V injuries, to reduce the risk of peripancreatic collections and related complications. Multicenter and larger-sample-size studies are required.

## Figures and Tables

**Table 1:** Demographics, clinical features, associated injuries, and complications in surgically managed patients with severe pancreatic injury.

Variables	Value	Variables	Value
**Age in years (SD)**	29.5 (8.5)	**Severity of injury**	
**Males**	11 (84.6%)	Pancreatic AIS	3.9 (0.9)
**Trauma type**		Chest AIS	2.7 (0.5)
Blunt	12 (92.3%)	Abdomen AIS	3.8 (1.1)
Penetrating	1 (7.7%)	Spine AIS	2.0 (0.0)
**SBP**	116.3 (10.7)	Injury severity score	21.2 (8.3)
**DBP**	76.5 (10.6)	RTS	7.8 (0.3)
**Pulse**	93.4 (15.4)	**Pancreatic injury**	
**GCS; median (range)**	15 (12–15)	Pancreatic laceration	4 (30.8%)
**Serum amylase**	175 (29–1003)	Pancreatic head	5 (38.5%)
**Serum lipase**	394 (42–1527)	Complete Transection	3 (23.1%)
**FAST**	9 (69.2%)	Pancreatic body	3 (23.1%)
**Associated injuries**		**Pancreatic complications**	
Chest injury	5 (38.5%)	Peripancreatic collection	9 (69.2%)
Pulmonary contusions	1 (7.7%)	Pseudocyst	1 (7.7%)
Hemothorax	2 (15.4%)	Pancreatic fistula	4 (30.8%)
Rib fracture	5 (38.5%)	Pancreatic abscess	2 (15.4%)
Spleen	6 (46.2%)	Pancreatic necrosis	1 (7.7%)
Liver	8 (61.5%)	Postoperative ileus	8 (61.5%)
Small bowel	3 (23.1%)	Intra-abdominal infection	8 (61.5%)
Colon	3 (23.1%)	Small-bowel obstruction	4 (30.8%)
Mesentery	3 (23.1%)	Enterocutaneous fistula	1 (7.7%)
Kidney	3 (0.0%)	Anastomotic leak	3 (23.1%)
Inferior vena cava	1 (7.7%)	Diabetes mellitus	2 (15.4%)
Retroperitoneal hematoma	2 (15.4%)	Wound infection	4 (30.8%)
Spine injury	3 (23.1%)	Wound dehiscence	2 (15.4%)
		Intra-abdominal bleeding	1 (7.7%)

Standard deviation (SD). GCS: Glasgow Coma Scale; FAST, Focused Assessment with Sonography in Trauma; SBP, systolic blood pressure; DBP, diastolic blood pressure.

**Table 2:** Summary of individual cases (n = 13).

Case	Age/Sex	Mechanism/Type	AAST Grade	Associated Injuries	Surgical Intervention	Complications	Outcome
1	28/M	MVC (Blunt)	V	Hemothorax, rib fracture, splenic, colonic, mesenteric and renal injury, retroperitoneal hematoma, spinal injury	Distal pancreatectomy + splenectomy; left nephrectomy, left hemicolectomy, bowel resection; 3 relaparotomies	Peripancreatic collections, pancreatic fistula, pancreatic abscess, intra-abdominal infection, anastomotic leak, sepsis, AKI, coagulopathy	Alive; LOS 19 d, ICU 4 d
2	17/M	Pedestrian struck (Blunt)	IV	Hemothorax, splenic injury	Distal pancreatectomy with spleen preservation; IR embolization of spleen	Peripancreatic collection, pseudocyst, post-prandial epigastric pain	Alive; LOS 25 d, ICU 7 d
3	19/M	Gunshot wound (Penetrating)	IV	Liver and IVC injury, retroperitoneal hematoma	Repair of pancreatic laceration; exploratory laparotomy	Peripancreatic collection, pancreatic abscess, intra-abdominal infection, wound infection, sepsis	Alive; LOS 31 d, ICU 2 d
4	39/M	MVC (Blunt)	V	Liver and colonic injury	Packing of pancreatic head; 3 relaparotomies	Peripancreatic collection, enterocutaneous fistula, anastomotic leak, wound infection/dehiscence, ARDS, postoperative ileus, intra-abdominal infection, sepsis	Alive; LOS 80 d, ICU 27 d (vent 19 d)
5	45/M	Self-inflicted (Blunt)	III	Splenic and renal injury	Distal pancreatectomy + splenectomy; chest tube; ERCP	Peripancreatic collection, main pancreatic duct injury, splenic artery thrombosis, pancreatic fistula, SBO, DM (pancreatic insufficiency), pulmonary embolism, pleural effusion, sepsis	Alive; LOS 62 d, ICU 1 d
6	36/M	Assault (Blunt)	V	Liver and small-bowel injury	Distal pancreatectomy + splenectomy	Peripancreatic collection, pancreatic fistula, postoperative ileus, sepsis	Alive; LOS 17 d, ICU 1 d
7	29/M	Fall (Blunt)	III	Rib fracture, splenic and liver injury	Distal pancreatectomy + splenectomy; chest tube; hepatic artery embolization	Peripancreatic collection, main pancreatic duct laceration, splenic vein thrombosis, DM (pancreatic insufficiency), postoperative ileus, intra-abdominal infection, sepsis	Alive; LOS 23 d, ICU 8 d (vent 1 d)
8	29/F	ATV collision (Blunt)	V	Rib fracture, small-bowel, mesenteric and renal injury, spinal injury	Packing only; 10 relaparotomies; tracheostomy	Pancreatic necrosis, anastomotic leak, SBO, wound dehiscence, pneumonia, postoperative ileus, intra-abdominal infection, sepsis, AKI	Died; LOS 23 d, ICU 23 d (vent 23 d)
9	40/M	Blunt force (“thud”) (Blunt)	IV	Rib fracture, splenic, liver and colonic injury	Distal pancreatectomy + splenectomy; chest tube; 10 relaparotomies; CT-guided drainage; ERCP	Pancreatic fistula, SBO, wound infection, intra-abdominal bleeding, pleural effusion, postoperative ileus, intra-abdominal infection, sepsis	Alive; LOS 107 d, ICU 36 d (vent 13 d)
10	28/M	Blunt force (“thud”) (Blunt)	III	Spinal injury	Distal pancreatectomy with spleen preservation; CT-guided drainage; ERCP	Peripancreatic collection, SBO, pleural effusion, postoperative ileus, intra-abdominal infection, sepsis	Alive; LOS 39 d, ICU 0 d
11	28/M	Motorcycle collision (Blunt)	III	Pulmonary contusion, rib fracture, liver injury	Subtotal pancreatectomy + splenectomy	Peripancreatic collection, sick euthyroid syndrome	Alive, discharged with drain; LOS 26 d, ICU 5 d
12	25/F	Explosion (Blunt)	III	Splenic and liver injury	Distal pancreatectomy + splenectomy; 1 relaparotomy	None reported	Alive; LOS 1 d, ICU 0 d
13	20/M	Fall (Blunt)	IV	Liver, small-bowel and mesenteric injury	Distal pancreatectomy with spleen preservation	Wound infection	Alive, discharged with drain; LOS 17 d, ICU 10 d (vent 2 d)

High-grade pancreatic injuries (AAST grade III–V). MVC = motor vehicle collision; ATV = all-terrain vehicle; IVC = inferior vena cava; SBO = small-bowel obstruction; DM = diabetes mellitus; AKI = acute kidney injury; ARDS = acute respiratory distress syndrome; LOS = length of stay; ICU = intensive care unit; vent = ventilator days.

**Table 3:** Complications and outcomes in surgically managed patients with severe pancreatic injury.

Variables	Value	Variables	Value
**Pancreatic resection type**		Blood transfusion	6 (46.2%)
Distal pancreatectomy and splenectomy	7 (53.8%)	**In-hospital complications**	
Distal pancreatectomy with spleen preservation	3 (23.1%)	Pneumonia	1 (7.7%)
Packing/repair	3 (23.1%)	Acute respiratory distress syndrome	1 (7.7%)
**Pancreatic complications**		Pulmonary embolism	1 (7.7%)
Peripancreatic fluid collection	10 (76.9%)	Pleural effusion	4 (30.8%)
Drain	1 (7.7%)	Sepsis	9 (69.2%)
None	2 (15.4%)	Acute kidney injury	2 (15.4%)
**Management of complication**		Coagulopathy	1 (7.7%)
CT-guided drainage	4 (36.4%)	**Ventilatory days**	8 (1–23)
CT-guided drainage + ERCP	1 (9.1%)	**Hospital length of stay**	25 (1–107)
Conservative	2 (18.2%)	**ICU length of stay**	7 (1–36)
Surgery + CT-guided drainage + ERCP	1 (9.1%)	**Mortality**	1 (7.7%)
Surgery	1 (9.1%)	**Discharge condition**	
ERCP	1 (9.1%)	Stable	10 (76.9%)
Relook laparotomy	1 (9.1%)	Discharge with drain	3 (23.1%)
**Octreotide during hospital stay**	6 (46.2%)		
**Chest tube insertion**	4 (30.8%)		
**Exploratory laparotomy**	9 (69.2%)		
**No. of repeat laparotomies**	3 (1–10)		

CT, computed tomography; ERCP, endoscopic retrograde cholangiopancreatography.

**Table 4:** Comparison of the higher injury grades.

Variables	Grade III (n = 5; 38.5%)	Grade IV (n = 4; 30.8%)	Grade V (n = 4; 30.8%)
**Age in years (SD)**	31.0 (7.9)	24.0 (10.7)	33.0 (5.4)
**Males**	4 (80.0%)	4 (100%)	3 (75.0%)
**Associated injuries**			
Chest injury	1 (20.0%)	2 (50.0%)	5 (50.0%)
Spleen	3 (60.0%)	2 (50.0%)	1 (25.0%)
Liver	3 (60.0%)	3 (75.0%)	2 (50.0%)
Small bowel	0 (0.0%)	1 (25.0%)	2 (50.0%)
**Abdomen AIS**	2.6 (0.5)	4.0 (0.0)	5.0 (0.0)
**Injury severity score**	14.6 (4.3)	19.5 (4.7)	31.0 (5.0)
**Revised trauma score**	7.8 (0.0)	7.8 (0.0)	7.6 (0.5)
**Glasgow coma scale at ED**	15 (15–15)	15 (15–15)	15 (12–15)
**Serum amylase value**	71 (63–1003)	207 (29–332)	247 (81–485)
**Serum lipase value**	361 (42–1441)	415 (86–445)	772 (223–1527)
**Pancreatic resection type**			
Distal pancreatectomy and splenectomy	4 (80.0%)	1 (25.0%)	2 (50.0%)
Distal pancreatectomy with spleen preservation	1 (20.0%)	2 (50.0%)	0 (0.0%)
Packing/repair	0 (0.0%)	1 (25.0%)	2 (50.0%)
**Pancreatic complications**			
Peripancreatic collection	3 (60.0%)	3 (75.0%)	3 (75.0%)
Pancreatic fistula	1 (20.0%)	1 (25.0%)	2 (50.0%)
Pancreatic abscess	0 (0.0%)	1 (25.0%)	1 (25.0%)
Pancreatic necrosis	0 (0.0%)	0 (0.0%)	1 (25.0%)
**Management of complication**			
CT-guided drainage	1 (25.0%)	1 (33.3%)	2 (50.0%)
CT-guided drainage + ERCP	1 (25.0%)	0 (0.0%)	0 (0.0%)
Conservative	1 (25.0%)	1 (33.3%)	0 (0.0%)
Surgery + CT-guided drainage + ERCP	0 (0.0%)	1 (33.3%)	0 (0.0%)
Surgery	0 (0.0%)	0 (0.0%)	1 (25.0%)
ERCP	1 (25.0%)	0 (0.0%)	0 (0.0%)
Relook Laparotomy	0 (0.0%)	0 (0.0%)	1 (25.0%)
**Exploratory laparotomy**	3 (60.0%)	3 (75.0%)	3 (75.0%)
**Blood transfusion**	2 (40.0%)	1 (25.0%)	3 (75.0%)
**Sepsis**	3 (60.0%)	2 (50.0%)	4 (100%)
**Hospital length of stay**	26 (1–62)	28 (17–107)	21 (17–80)
**ICU length of stay**	5 (1–8)	8.5 (2–36)	13.5 (1–27)
**Mortality**	0 (0.0%)	0 (0.0%)	1 (25.0%)

## Data Availability

All data were given in the tables and figures. It can be obtained from the PI upon reasonable request and signing data sharing agreement.

## Abbreviations

WTA	Western Trauma Association
AIS	Abbreviated injury score
ISS	Injury severity score
GCS	Glasgow coma scale
HGPI	high-grade pancreatic injuries
